# Are aerosol control devices effective in preventing the spread of dental aerosol?

**DOI:** 10.7717/peerj.13714

**Published:** 2022-07-13

**Authors:** Elif Seher Böke, Ali Keleş, Cangül Keskin, Yeliz Tanrıverdi Çaycı, Tugba Turk

**Affiliations:** 1 Department of Endodontics, Faculty of Dentistry, Ondokuz Mayis University, Samsun, Turkey; 2 Department of Medical Microbiology, Faculty of Medicine, Ondokuz Mayis University, Samsun, Turkey; 3 Deapartment of Endodontics, Faculty of Dentistry, Ege University, İzmir, Türkiye

**Keywords:** Aerosol, COVID-19, Endodontics, *Enterococcus faecalis*, Dentistry, Aerosol control devices

## Abstract

**Background:**

In dental clinics, aerosols produced from dental instruments have become a matter of concern following breakout of coronavirus disease 19 (COVID-19) evolving into a pandemic. This study compared aerosol reduction systems and in terms of their ability to reduce Enterococcus faecalis (*E. faecalis*) contaminated aerosol in a simulated dental office set-up.

**Methods:**

Closed clinic model with manikin and mandibular molar typodont was simulated. For 10 min, the air and water dispersed by the rotating bur mounted on an aerator was contaminated by pouring the suspension containing 1–3 × 10^8^ CFU/mL *E. faecalis* directly on the bur. During and after the procedures, the air within the cabin was also sampled. CFU count was recorded and scored. The mean CFU scores obtained from agar plate count and air sampling device was compared using Kruskal–Wallis H test among groups with 5% significance threshold.

**Results:**

The use of WS Aerosol Defender device led to greater CFU scores on the agars levelled to patient’s chest compared to other directions (*p* = 0.001). Combined use of VacStation and WS Aerosol Defender resulted in significantly decreased CFU score in the air samples compared to experimental and positive control groups (*p* = 0 < 0.05).

**Conclusions:**

Although the devices prevented the spread of aerosol around the patient to some extent, they could not completely eliminate the contaminated aerosol load in the cabin environment.

## Introduction

Aerosols are airborne liquid and solid particles that could be responsible for the transfer of microorganisms through the air (Zemouri et al., 2017). In dental clinics, aerosols produced from dental instruments such as ultrasonic devices, aerators, micromotors, and air/water syringes have become a matter of concern following the breakout of coronavirus disease 19 (COVID-19) evolving into a pandemic (Leggat & Kedjarune, 2001; Nagraj et al., 2020). Aerosol occurrence cannot be prevented, and moreover, aerosol contaminated with microorganisms can suspend in the air and spread over distances of meters, increasing the risk of cross-infection (Volgenant & De Soet, 2018; Rautemaa et al., 2006). A previous case series showed that dental treatment, including the use of ultrasonic devices, resulted in bioaerosols containing the viruses from the patient with active lesions and cross-infection of the dentists and dental hygienists (Browning & McCarthy, 2012). Therefore, in a dental clinic set up, patients and healthcare workers are in the high-risk group for infection transmission (Kadaifciler & Cotuk, 2014; Szymańska, 2005; Borella et al., 2008).

COVID-19 is an infectious disease caused by the widespread transmission of the severe acute respiratory coronavirus 2 (SARS-CoV-2), which shows a particle size of 0.5–10 µm with a very high rate of spread of the infection through aerosol transmission (Jarvis, 2020). As the most common transmission route is through inhalation of contaminated aerosols and droplets, using the correct personal protective equipment, including gowns, masks and goggles, are critical for protection. Surgical masks are not effective in protecting against droplet transmitted viruses such as influenza, SARS-CoV, or MERS-CoV (Umer, Haji & Zafar, 2020). Due to the existence of SARS-CoV-2 infected patients who do not show any obvious symptoms, it is necessary to take precautions as if each patient was positive in an open dental clinic to protect healthcare workers and other patients if early screening is not possible or in case of dental emergencies (Jiang et al., 2021). The changes in the dental procedure guidelines during different phases of the pandemic also have negative economic implications by affecting the number of patients receiving treatment on a workday and increasing the costs of clinical equipment (Lo Nigro et al., 2020). Therefore, the elimination of aerosol emerged as a crucial precaution to provide a safer patient circulation.

The use of mouthwashes before the dental procedures have been considered to reduce the number of microorganisms in the spreading aerosol (Marui et al., 2019). Pharmacotherapy was also recommended to reduce oral secretions producing aerosols (Mohammad Sadeghi et al., 2020). Novel devices to limit or prevent aerosol spread are also commercialized, and some of them have been shown to reduce the spread effectively in *in vitro* settings  (Teichert-Filho et al., 2020; Shahdad et al., 2020; Nulty et al., 2020). Since some of these devices are extraoral and some are intraoral, their combined use can also be tried to minimize aerosol distribution. VacStation (Eighteeth, China) is an extraoral aerosol vacuum device using high-efficiency particulate air (HEPA) filter and ultraviolet light with the claim to trap viruses and germs bigger than 0.3 µm with 99.97% efficiency. Water Saliva Aerosol Defender (WS Aerosol Defender; Cefla, Italy) has a sliding suction pipe placed around the patient’s mouth and has been developed following the breakout of the COVID-19 pandemic to reduce aerosol diffusion (Gandolfi, Zamparini & Spinelli, 2020).

These and similar devices have become popular as a preventative measure for the continuity of dental care since the beginning of the pandemic. However, the efficacy WS Aerosol Defender or its combined use with extraoral vacuum devices has not yet been evaluated, yet. The aim of the present study was to evaluate the efficacy of different aerosol reduction systems and their combinations to prevent the spread of *Enterococcus faecalis* (*E. faecalis*) contaminated aerosol generated in a simulated closed dental procedure on a manikin. The null hypothesis was that no significant difference was found in different vacuum systems and control groups in terms of the number of colony forming units (CFU) that will form on the agar mediums places on different directions and air samplings.

## Methods

The sample size was determined by selecting the F-test family ANOVA (fixed effects, omnibus, one-way) using the effect size calculated from a previous study (Holloman et al., 2015) with the alpha type error of 0.05 and a beta power of 0.80 (G*Power 3.1 for Mac; Heinrich Heine, Universitat Dusseldorf, Dusseldorf, Germany). The minimum sample size required to observe the same effect size was indicated as 20 measurements per group.

A suspension of *E. faecalis* (ATCC 25922; American Type Culture Collection, Manassas, VA, USA) was cultivated on 5% sheep blood agar and checked for reproductive purity after incubation. Then, a suspension of 0.5 McFarland standard was prepared to contain 1–3 × 10^8^ CFU/mL *E. faecalis*.

A special closed cabin with 3 × 3 × 3.5 m dimensions containing a dental unit in its center and a window in the opposite wall of the dental unit was used for experiments to provide a standardization among different experimental groups. A phantom mannikin (Frasaco GmbH, Tettnang, Germany) was placed on the dental unit in a standardized position as the mandibular occlusal plane was parallel to the floor. The left mandibular first molar typodont of the phantom model was isolated with rubber dam (Cerkamed, Wojciech, Poland), which was disposed of and renewed after each experimental procedure. Between each experiment, the window was opened, and ultra-low volume device containing hydrogen peroxide was used for disinfectant fogging. Then, the cabin’s surfaces, the manikin, and the dental unit were cleaned and disinfected with a hospital disinfectant containing 90% alcohol and hydrogen peroxide. The handpiece and metal pieces of rubber dam set was heat sterilized (at 134 °C for 75 min), and the efficacy of the sterilization process was controlled with chemical indicators (AXIS, Izmir, Turkey). Finally, the cabin was ventilated for half an hour. Following disinfection and ventilation, an open agar medium was placed on a designated standard area, and the intra-cabin air was sampled (AIR IDEAL, Biomeriux, France) for 15 min to validate the efficacy of disinfection. Then the agar plate was incubated at 37 °C for 24 h, and if there was no growth, the cabinet was considered disinfected, and the next experimental stage was initiated.

A dental turbine handpiece (W&H Alegra, Austria) with an attached diamond fissure bur with a green band (Diamant, Langenhagen, Germany) was operated passively on the isolated tooth for 10-min. Sterile distilled water was used in the unit. 1–3 ×10^8^ CFU/mL of *E. faecalis* extract was slowly poured onto the rotating fissure bur using a 10 mL dental syringe in 10 min.

The experimental groups were constructed as follows;

### VacStation group

VacStation (Eighteeth, China) was placed next to the cuspidor, which was on the left side of the dental unit. The funnel part of the device was positioned in front of the manikin at a distance of 15 cm from its mouth. The device was operated at the maximum suction power for 15 min in accordance with the instructions of the manufacturer; 10 min during aerosol formation procedure and during the following 5 min period. Agars positioned around the manikin head were then collected, but air sampling lasted for another 15 min reaching a total sampling time of 30 min.

### WS Aerosol Defender group

WS Aerosol Defender (De Cefla, Italy) was fixed around the mouth, positioned according to the left mandibular molar area, and connected to the surgical suction according to the instructions of the manufacturer for 10 min during the aerosol formation procedure and the following 5 min period with the maximum suction power of the dental unit. After each test, the WS Aerosol Defender system was disassembled, and the parts were washed and heat sterilized (at 121°C for 20 min). Sterilization validity was checked with chemical indicators (AXIS, Izmir, Turkey).

### VacStation + WS Aerosol Defender group

Simultaneous use of the VacStation and WS Aerosol Defender. First, the WS Aerosol Defender was attached to the surgical suction of the dental unit and positioned for the left mandibular molar area as described for the Group 2. Then, the VacStation was operated as described for the Group 1. Both devices were used for 15 min, 10 min during the aerosol formation, and the next 5 min.

### Extraoral barrier group

**Figure 1:**
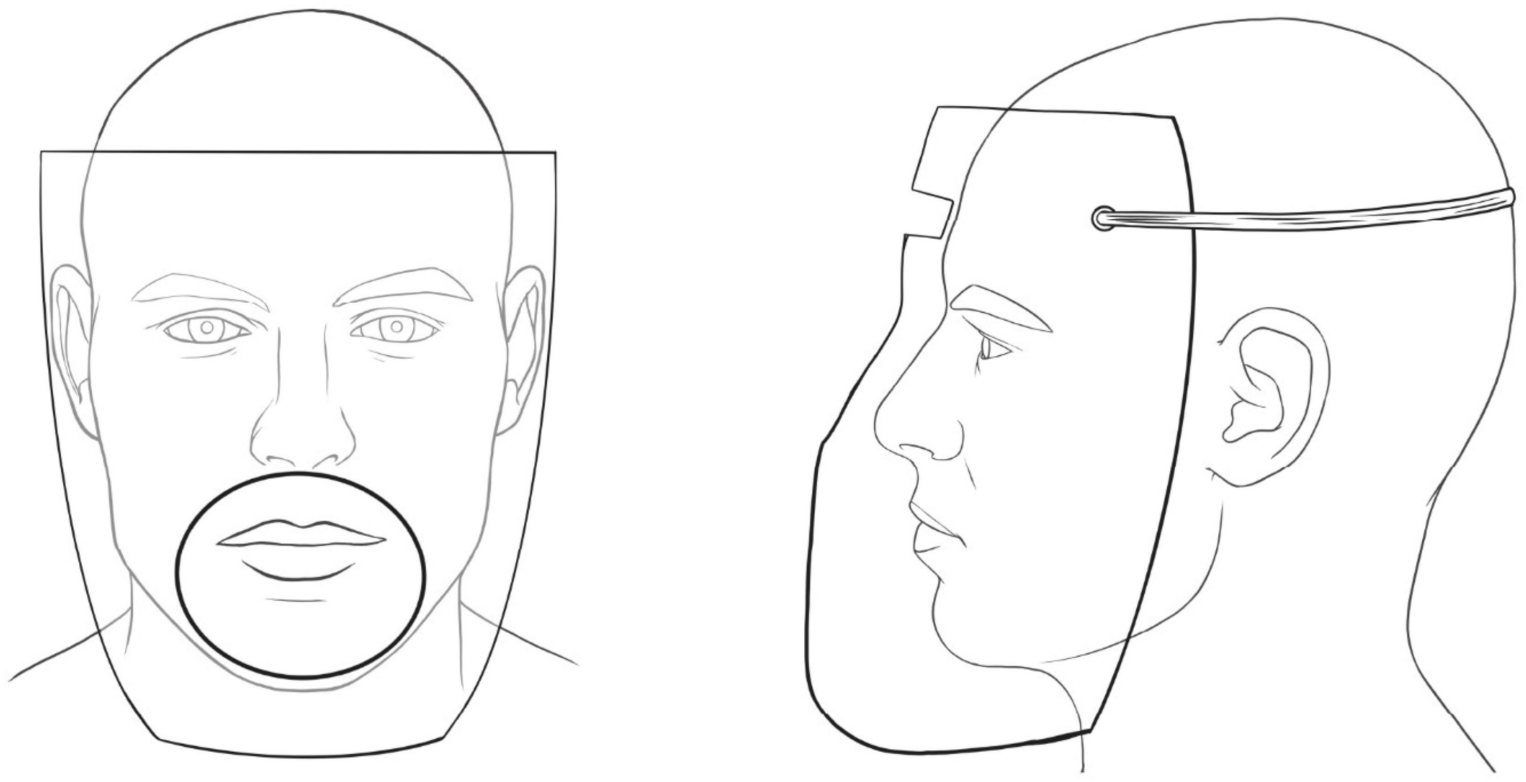
Extraoral protective barrier. The front and side view of the extraoral protective barrier with a circle shaped opening with 10 cm diameter and with rubbers to attach the head.

An extraoral protective shield produced from acetate in the thermal vacuum device by pressing over the mold suitable to the manikin’s face was used to cover the face (Fig. 1). A circle-shaped opening with a 10 cm diameter, where the handpiece could pass and reach the relevant tooth, was prepared. The barrier was attached to the manikin’s head with rubbers. The total sampling time was applied as described above, and then the barrier was removed and disposed of.

### Positive control group

No preventive device was used during the contaminated aerosol production period of 10 min and the following 5 min period. The control procedure was repeated between each experiment conducted in groups 1 to 4 to validate bacterial viability. The total sampling time was applied as described for the remaining groups as 15 min.

### Negative control group

This group includes the aerosol generation as described above without contamination with *E. faecalis* with no use of suction or vacuum devices for 10 min and a total sampling of 15 min.

**Figure 2:**
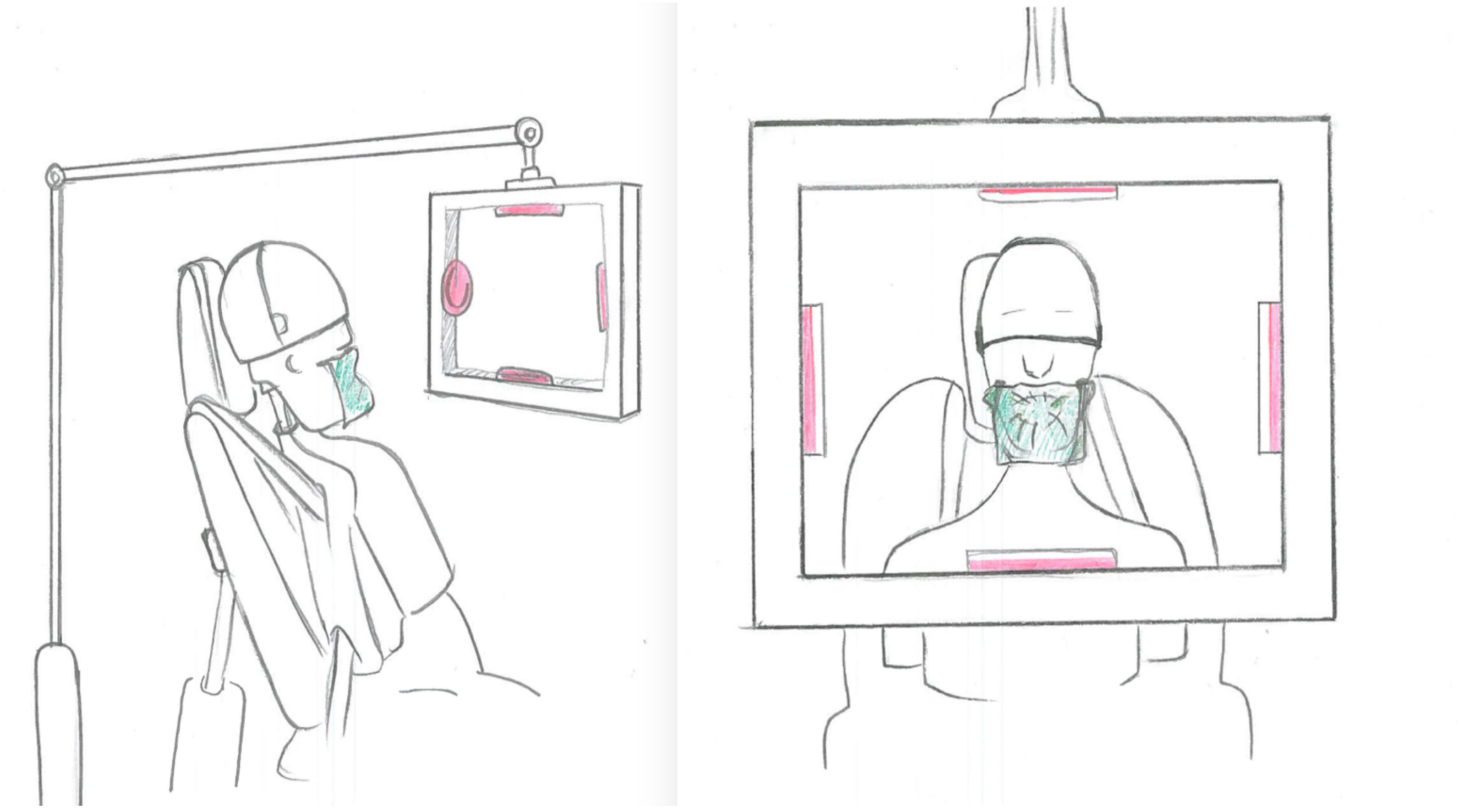
Experimental set up. The schematic representation of the experimental set up including the simulated patient and the placement of the agar plates.

During the experiments, four agar plates were placed in standardized positions within a custom-made carrier located at 30 cm from the manikin containing agar plates on the right (clinician’s side), left (dental assistant’s side), right at the chest level of the manikin (patient side) and above of the manikin’s head (Fig. 2). The air within the cabin was also sampled for 30-min during the experiments using the air sampling device (AIR IDEAL) placed on the unit table 1 m away from the manikin and its level. The bacterial load inside the cabin at each experimental procedure was calculated.

**Table 1:** The descriptive statistics and score distribution for the contaminated aerosol detected by air sampling device mounted on the unit table for the experimental and positive control groups.

**Group**	**Mean (Std)**	**IQR (25th)**	**Median**	**IQR (75th)**	**Min–Max**
**VacStation**	1.75 (1.25)^a^	0	2	3	0–3
**WS Aerosol Defender**	1.95 (0.88)^a^	1	2	3	0–3
**Extraoral Barrier**	1.90 (1.25)^a^	0.75	2	3	0–3
**VacStation**+**WS Aerosol Defender**	0.65 (0.74)^b^	0	0.5	1	0-2
**Positive control**	2.40 (0.51)^a^	2	2	3	2–3
**CFU Scores**					
**Group**	**No growth (0)**	**0–10 (1)**	**10–50 (2)**	**50–100 (3)**	**>100 (4)**
**VacStation**	6	0	7	7	0
**WS Aerosol Defender**	1	5	8	6	0
**Extraoral Barrier**	5	1	5	9	0
**VacStation**+**WS Aerosol Defender**	10	7	3	0	0
**Positive control**	0	0	6	4	0

**Notes.**

Different superscript letters in the same column indicate significant difference according to Kruskal–Wallis H test (*p* < .05).

Then the agar plates were incubated at 37 °C for 24 h. In the presence of growth, Gram staining, catalase reaction, and oxidase tests were performed, and preliminary identification of the growing microorganisms was performed. The microorganism type and CFU numbers were determined, and genus and species identification was made using Vitek MS (Biomeriux, France). The number of CFUs detected on each agar plate according to each direction and the total CFUs were counted, and scored as described:

Score 0: No growth was observed

Score 1: 0–10 CFU/mL

Score 2: 10-50 CFU/mL

Score 3: 50–100 CFU/mL

Score 4: >100 CFU/mL

The comparison of the air sampling and CFU count scores according to the experimental groups and directions was performed using Kruskal–Wallis H test with a 5% significance threshold (SPSS, IBM, Chicago, IL, USA).

## Results

**Figure 3:**
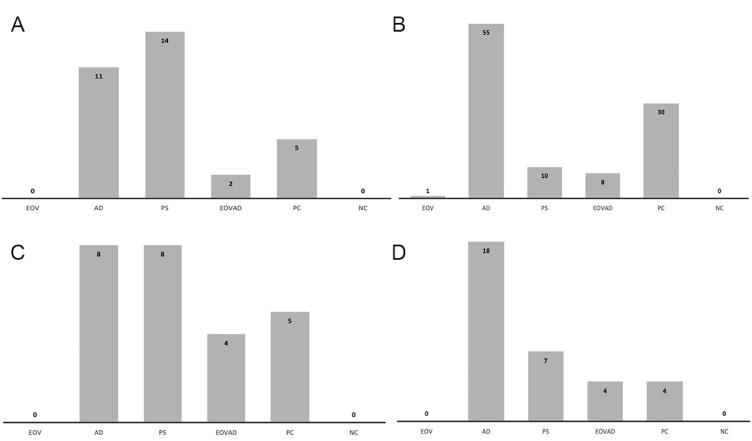
Histogram showing the sum of the mean CFU scores of the experimental groups with control groups. Histogram showing the sum of the mean CFU scores of the experimental groups with the positive (PC) and negative control (NC) groups according to the directions: (A) assistant side, (B) patient side, (C) dentist side, (D) above the patient. VS:VacStation, AD: WS Aerosol Defender group, EB: Extraoral barrier group, VSAD VacStation + WS Aerosol Defender group.

In the positive control group in which no device was used against bacteria-contaminated aerosol spread, the mean number of CFU was significantly higher in agar plates positioned at the patient’s chest level compared to those positioned at the right (clinician) and left (dental assistant) and above the manikin’s head. (*p* =0.001). No growth was detected in the negative controls. Figure 3 represents the the sum of the mean CFU scores of the experimental groups with the positive and negative control groups for each direction.

In VacStation group, none of the agar plates placed on the right (clinician), left (dental assistant), and above the manikin’s head showed bacterial growth. Among the agar plates placed on the manikin’s chest level, only one plate was scored to show 0–10 CFU/mL while the remaining showed no growth. VacStation significantly reduced contaminated aerosol on the patient’s chest level similar to the extraoral barrier or its combined use with WS Aerosol Defender, compared to the positive control (*p* = 0.001). No significant difference regarding the mean scoring among different directions was observed when the VacStation was used (*p* = 1.00). Air sampling data was similar to that of the positive control group (*p* = 0.184) (Table 1).

In WS Aerosol Defender group, the mean CFU score was significantly higher in the agar plates placed on the patient’s chest level than in other directions (*p* = 0.001). In this group, the agar plates placed above the patient were significantly more contaminated than VacStation and the combined group (*p* = 0.001). Air sampling data for the WS Aerosol Defender was similar to those of VacStation (*p* = 0.706) and positive control groups (*p* = 0.308) (Table 1). However, in the WS Aerosol Defender group, the agar plates positioned at the patient’s chest level and dental assistant side showed significantly higher scores compared to the dentist side and above the patient head side (*p* = 0.001). The agar plates positioned on the right (clinician) side showed similar scores in all experimental and positive control groups (*p* = 0.082).

Simultaneous use of the VacStation and the WS Aerosol Defender resulted in a significantly lower CFU count in the sampled air than in other groups and positive control (*p* < 0.05). The mean CFU count in agar plates showed no significant difference regarding their position (*p* > 0.05). In this group, CFU scores of the agar plates, irrespective of their position, were lower than the positive control group; however, it was only significant at agar plates placed on the patient’s chest level (*p* < 0.05).

In the extraoral barrier group, the mean CFU scores of the agar plates were similar irrespective of their position (*p* > 0.05). Furthermore, in the extraoral barrier group, CFU scores of agar plates from all positions were lower than the positive control group; however it was only significant at which were placed on the patient’s chest level (*p* < 0.05). Air sampling data were similar to groups 1, 2, and the positive control group (*p* > 0.05).

## Discussion

This study compared the efficacy of an independent extraoral vacuum device, an aerosol reduction tool attached to the dental unit, their combination and an extraoral barrier to prevent bacteria-contaminated aerosol spread and found statistically significant differences in terms of the device and direction of contaminated aerosol spread. From a clinical aspect, it should be noted that although none of the techniques eliminated the spread of aerosol from the environment, some methods were more efficient in reducing the contaminated aerosol. Therefore, the null hypothesis was rejected.

The most important advantage of the extraoral vacuum device VacStation that its efficacy does not depend on the dental unit and its portability. In the present study, the VacStation significantly reduced contaminated aerosol on the patient’s chest level compared to the positive control group. However, the actively sampled cabin air during and following the use of the VacStation showed similar CFU scores to those of the positive control group. The relatively shorter passive sampling duration (15 min) of the agar plates placed around the manikin could be inadequate to detect dispersed contaminated aerosol in this group or extraoral vacuum device was able to eliminate aerosol within a short range when used with the maximum power suction. The present study’s findings were in accordance with previous studies using different extraoral vacuum aspirators despite the differences in device designs and evaluation methods (Shahdad et al., 2020; Noro et al., 1995; Teanpaisan, Taeporamaysamai & Rattanachone, 2001). Although using an extraoral vacuum device reduces the dispersed aerosol, ventilation of the cabin after aerosol-generating dental procedures still seems to be the most critical precaution in clinical scenarios where the extraoral vacuum device was used alone.

The sampled cabin air showed similar scores in the VacStation, the extraoral barrier, and the positive control groups. Since the WS Aerosol Defender is attached to the surgical suction of the dental unit, its vacuum power is dependent on the dental unit’s suction power. Users should routinely check the power of the unit for blockage or backflows that might limit its performance. Therefore, the present study’s findings should be interpreted considering the capacity of the dental unit where the device was attached, without generalizing them for each dental clinic set-up. Further studies using different clinical scenarios are warranted to evaluate the efficacy of WS Aerosol Defender device more thoroughly.

The use of personal protective equipment during the aerosol-producing dental procedures is essential for safety since it is widely known that the contaminated aerosol exposes the face, hair, and cloths of dental staff who work in close proximity to the patient (Bennett, Fulford & Walker, 2000; Centers for Disease Control and Prevention, 0000; Li et al., 2004). In the present study, the CFU scores obtained from each group were also compared according to the directions. The present study’s findings also emphasized the importance of personal protective equipment since no prevention method could be able to prevent contamination around the manikin. On the clinician’s side, all groups reached similar CFU scores; however, on the dental assistant’s side, WS Aerosol Defender resulted in significantly higher scores than the VacStation, the extraoral barrier, and its use combined with VacStation. The difference in the dispersion of the contaminated aerosol between the clinician and the dental assistant’s sides could occur due to selecting the left mandibular molar typodont. However, a recent study reported a similar finding that the assistant’s arm and chest were the most contaminated in a closed dental set-up, irrespective of the operation side (Shahdad et al., 2020). In the present set-up, readers still should be reminded that the present study’s findings are strongly related to the position of the left mandibular molar typodont and should be interpreted with caution that the aerosol dispersion for a maxillary incisor or right mandibular premolar would be different.

The highest CFU scores were seen on the agars placed at the patient’s chest level in the WS Aerosol Defender and the positive control groups. A recent study demonstrated splatter contamination on the dental staff and suggested wearing longer visors and gowns to cover the head, neck, and chest area (Shahdad et al., 2020). The dental staff disposes of their protective equipment in the workspace. However, the patient usually wears a disposable apron of various sizes and materials to cover the chest area and leaves the office with possibly contaminated clothes. This study showed that it might be suggested to use larger aprons to completely cover the patient’s neck, shoulders, and chest and have the patient wear a surgical cap and gown. Contamination of the patient is especially important for open-plan clinics where multiple patients are treated and shared X-ray rooms during treatment. Although the most crucial precaution will be the pre-screen tests before the procedures, rare false-negative results may still occur (Cochrane COVID-19 Diagnostic Test Accuracy Group, 2021). Even if an unrecognized, asymptomatic or pre-symptomatic patient wearing masks could pose a threat with their aerosol contaminated clothing or hair for spread after leaving the dental office aerosol generating dental procedures. Although no evidence proves a transmission in this way, being meticulous at this pandemic stage will benefit public health since the unrecognized, asymptomatic or pre-symptomatic patients have been regarded as a source of infection (Kimball et al., 2020). Therefore, in addition to pre-procedure scans, personal protective equipment to protect the patient’s hair and clothes from aerosol may be considered.

In this study, a disposable protective barrier was used to cover the patient’s face without interfering the clinician’s operation. Since the barrier did not have any vacuuming, its effect differed according to the location of agar plates. It showed a similar effect to the positive control in spreading of the contaminated aerosol on the right and left sides. The contamination at the patient’s chest level was significantly less than the WS Aerosol Defender and the positive control groups. However, the agar plates above the patient’s head showed greater CFU scores in this group.

The maximum efficacy in reducing bacterial load in the cabin air was achieved when the extraoral vacuum device and WS Aerosol Defender were combined. Half of the cabin air samples showed no growth in this group, which performed significantly superior to the remaining groups, including the positive control. The agar plate scores were similar to those of the extraoral vacuum and the extraoral barrier groups irrespective of the directions. Thereby, in this study, none of the techniques could prevent aerosol spread completely. The use of a phantom model without simulating the saliva and oral microbiome is a limitation and further confirmatory studies on patients are warranted.

## Conclusions

Within the limitations of the present study, none of the techniques were able to eliminate dental aerosol spread and the microbial load in the closed cabin model after aerosol production for 10 min of working time of a dental turbine in the left mandibular molar area. The use of VacStation lowered the aerosol spread at patient’s chest level, however, sampled cabin air was contaminated as the positive control group. WS Aerosol Defender resulted in a greater aerosol spread on patient’s chest area compared to the use of the VacStation and the extraoral barrier. In contrast, its combined use with VacStation significantly decreased the CFU score in the air samples.

## Supplemental Information

10.7717/peerj.13714/supp-1 Data S1 Raw Data CFU scores of each sample from each group.

## Additional Information and Declarations

### Data Availability

The following information was supplied regarding data availability: The raw measurements are available in the Supplementary File.
